# Key Role of Deep Orbitals in the d_*x*^2^–*y*^2^_–d_3*z*^2^–*r*^2^_ Gap in Tetragonal Complexes and 10*Dq*

**DOI:** 10.1021/acs.jpca.0c11609

**Published:** 2021-03-16

**Authors:** J. A. Aramburu, M. Moreno

**Affiliations:** Departamento de Ciencias de la Tierra y Física de la Materia Condensada, Universidad de Cantabria, Avenida de los Castros s/n, 39005 Santander, Spain

## Abstract

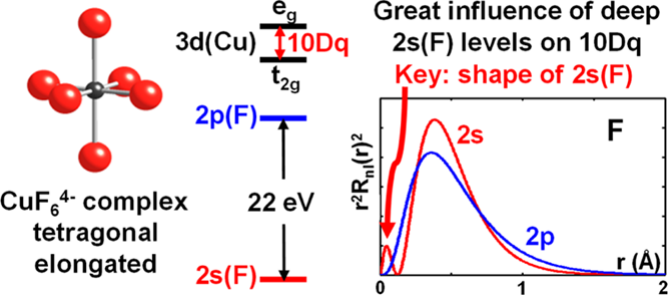

Using first-principles calculations, we show that the origin of the intrinsic a_1g_(∼3*z*^2^ – *r*^2^)–b_1g_(∼*x*^2^ – *y*^2^) splitting, Δ_int_, in tetragonal transition-metal complexes and the variations of the cubic field splitting, 10*Dq*, with the metal–ligand distance, *R*, are much more subtle than commonly thought. As a main novelty, the key role played by covalent bonding with deep valence ligand levels and thus the inadequacy of too simple models often used for the present goal is stressed. Taking as a guide the isolated *D*_4*h*_ CuF_6_^4–^ complex, it is proved that Δ_int_ essentially arises from bonding with deep 2s(F) orbitals despite them lying ∼23 eV below 2p(F) orbitals. This conclusion, although surprising, is also supported by results on octahedral fluoride complexes where the contribution to 10*Dq* splitting from bonding with 2s(F) orbitals is behind its strong *R* dependence, stressing that explanations based on the crystal-field approach are simply meaningless.

## 1 Introduction

A great deal of research is currently focused on transition metal (TM) compounds due to their potential technological interest, witnessed in lasers^1^ based on Al_2_O_3_:Ti^3+^ or BeAl_2_O_4_:Cr^3+^ or devices using manganites.^2^ Among insulating TM materials, particular attention is paid to those containing Cu^2+^ ions. Aside from the interest on La_2_CuO_4_, the parent compound of high-*T*_c_ superconducting cuprates,^3,4^ much work is done on Cu^2+^ hybrid perovskites^5−9^ currently employed in several devices and on Tutton salts^10−13^ containing Cu^2+^ due to their potential application in the study of enzymes. In addition, some Cu^2+^ compounds are responsible for the color of historical pigments^14^ or the stained glasses of medieval gothic architecture.^15^

In insulating TM compounds, active electrons are essentially confined in the MX_*N*_ complex formed by the TM cation, M, and the *N* ligands. For this reason, a deep insight into the covalent bonding inside the MX_*N*_ unit is crucial for understanding the actual origin of optical and magnetic properties of such compounds following the way started by the pioneering work by Sugano and Shulman.^16,17^ The present work is just addressed to prove that subtleties in chemical bonding can play a crucial role for reaching such a goal. Efforts are particularly focused to explain the origin of the dependence on the metal–ligand distances of two relevant splittings of the antibonding orbitals with a mainly d character in MX_6_ complexes: (1) the splitting 10*Dq* between e_g_(∼3*z*^2^ – *r*^2^, *x*^2^ – *y*^2^) and t_2g_(∼*xy*, *xz*, *yz*) levels in octahedral *O*_*h*_ complexes and (2) the splitting Δ between a_1g_(∼3*z*^2^ – *r*^2^) and b_1g_(∼*x*^2^ – *y*^2^) levels in tetragonal *D*_4*h*_ MX_6_ units.

For reaching these objectives, the analysis of first-principles calculations and available experimental data is crucial. Indeed, models that use fitting parameters hardly allow one to know the actual microscopic origin of phenomena.^18−20^ For this reason, rough approximations such as the superposition^21^ or the angular overlap^22^ models together with those based on the crystal field (CF) approach are meaningless for the present goal.

In a first step, the present work explores the influence of covalent bonding upon the splitting Δ in tetragonal MX_6_ units. Positive Δ values mean in this work that a_1g_(∼3*z*^2^ – *r*^2^) has a higher energy than b_1g_(∼*x*^2^ – *y*^2^). For clarifying the main ideas, the tetragonal CuF_6_^4–^ complex is taken as a guide throughout the present work as Δ has been determined for several compounds containing such a complex. It should be noted here that optical excitations do also depend on the internal electric field induced by the rest of the lattice ions upon the electrons confined in the complex^23−26^ and thus there is a contribution to Δ not related to the chemical bonding in the complex.

It is worth noting now that the gap between 2p(F) and 2s(F) valence orbitals of free F atom^27−29^ is about 23 eV. Accordingly, it could be expected that Δ is much more influenced by the covalent bonding with shallow 2p(F) than with deep 2s(F) orbitals. We prove in this work that such a guess is not correct as the reality is certainly more subtle.

Tetragonal complexes are observed for Cu^2+^-doped cubic lattices^30−33^ as a result of the so-called static Jahn–Teller effect,^34−36^ a phenomenon ultimately due to the unavoidable presence of random strains in any real crystal.^34^ Tetragonal CuF_6_^4–^ units are also formed in Cu^2+^-doped K_2_ZnF_4_ or Ba_2_ZnF_6_-layered perovskites though there is no Jahn–Teller effect as the a_1g_(∼3*z*^2^ – *r*^2^) and b_1g_(∼*x*^2^ – *y*^2^) levels are not degenerate following the *tetragonal* symmetry of the host lattice.^24^ Accordingly, the theory describing the Jahn–Teller effect^34,35^ cannot, in general, be transferred^4,9,24^ to understand pure layered compounds such as the orthorhombic K_2_CuF_4_ or Cs_2_AgF_4_.^37,38^ Nevertheless, the Jahn–Teller framework is still surprisingly applied^39−41^ to d^9^ ions under tetragonal or lower symmetries.

*Compressed* tetragonal CuF_6_^4–^ units are formed in KAlCuF_6_ or CuFAsF_6_ pure compounds^42−45^ in addition to Cu^2+^-doped crystals.^24,46−48^ By contrast, in CuF_2_ or A_2_CuF_4_ (A = K, Na), the tetragonally compressed CuF_6_^4–^ units undergo an *additional* orthorhombic distortion, favored by the existence of adjacent complexes which share F^–^ ligands.^9,48,49^ An orthorhombic instability also takes place in copper Tutton salts^12^ and in NH_4_Cl:CuCl_4_(H_2_O)_2_^2–^.^50,51^

The interest in the Δ gap relies on the fact that it is often the lowest optical excitation of compounds^52^ with tetragonal MX_6_ units (M = Cu, Ag). Also, in superconductor oxocuprates, the transition temperature, *T*_c_, has been related to the magnitude of the Δ splitting.^53^

In a second step, the present work is devoted to clarify quantitatively the origin of the sensitivity of 10*Dq* to variations of the metal–ligand distance, *R*, in octahedral complexes. Experimentally, it has been found that 10*Dq* depends on *R*^–*t*^, where the exponent *t* is often found to be close to 5.^54^ By this reason, it is still claimed that the exponent *t* mainly comes from the CF contribution^40,55^ despite this approach leading to 10*Dq* values much smaller than experimental ones.^54^

Seeking to shed light on these issues, in addition to investigating the relation between covalent bonding and the splitting Δ, we have carried out first-principles calculations on tetragonal CuF_6_^4–^ units at different values of axial (*R*_ax_) and equatorial (*R*_eq_) metal–ligand distances. In this analysis, particular attention is paid to explore how the charge on ligands is modified by varying the *R*_ax_ and *R*_eq_ distances. In a further step, we analyze in octahedral complexes how the variations of chemical bonding with the metal–ligand distance, *R*, are quantitatively related to the exponent *t*.

This work is organized as follows. A brief account of computational tools is given in Section 2 while Section 3 first deals with the two contributions to optical transitions for a TM complex in an insulating compound: the intrinsic one associated with the isolated complex and the extrinsic one due to the internal electric field created by the rest of the lattice ions.^23,24^ That section also deals with the relation between the splitting, Δ, and the variation of charge on ligands. The main results of this work are discussed in Section 4. Special attention is paid in that section to clarify the different influences of bonding with 2p(F) and 2s(F) orbitals upon the splitting Δ (Section 4.1) of isolated CuF_6_^4–^ units and also the origin of the dependence of 10*Dq* on the metal–ligand distance for octahedral complexes (Section 4.2). For the sake of completeness, the reasons behind the similarities and differences between 2p(F) and 2s(F) orbitals are discussed in Section 4.3. Finally, the applicability of the present ideas to complexes involving Cl^–^, Br^–^, or O^2–^ as ligands is briefly dealt with in the last section.

## 2 Computational Methods

Ab initio density functional theory (DFT) calculations on *isolated* CuF_6_^4–^ complexes have been performed at fixed metal–ligand distances by means of the 2017.03 version of the Amsterdam density functional code.^69^ By means of this kind of calculations, we can already explore the dependence of the intrinsic contribution Δ_int_ to the Δ gap upon metal–ligand distances. In these DFT calculations, we have used the popular B3LYP hybrid functional (including 25% of Hartree–Fock exchange^70^) in the spin-restricted and spin-unrestricted Kohn–Sham formalism of the DFT and high-quality all-electron basis sets of triple-ζ plus polarization type. We have verified that similar results are obtained using other hybrid functionals such as the nonempirical PBE0 one.^71^

## 3 Influence of Covalent Bonding upon the Δ Splitting in Tetragonal Complexes: A General View

Although in insulating compounds containing TM cations, active electrons are localized in the MX_*N*_ complex, the optical properties cannot, in general, be explained considering only that isolated unit. Indeed, the localized electrons lying in the MX_*N*_ complex are *also* subject to the electric field, 
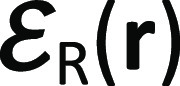
, created by the rest of the lattice ions, which usually has a perturbative character.^23^ By this reason, the energy, *E*, of an electronic transition can be divided in two contributions^24^
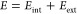
1where *E*_int_ is the intrinsic contribution to the *isolated* MX_*N*_ complex at equilibrium geometry while the extrinsic one, *E*_ext_, accounts for the effects of the internal electric field, 
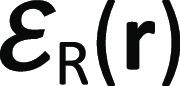
, upon the confined electrons. As an example, the intrinsic contribution for 10*Dq* in ruby and emerald^23,56^ is the same (10*Dq*_int_ = 2 eV), reflecting the identical Cr^3+^–O^2–^ distance (1.97 Å) in both gemstones.^23,57,58^ Thus, the difference between the red ruby and the green emerald simply arises from the distinct shape of the extrinsic 
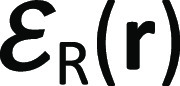
 field in the two gemstones, leading to the small corrections 10*Dq*_ext_ = 0.24 eV for ruby and 10*Dq*_ext_ = −0.05 eV for emerald.^23,56^ In the same vein, the color of the Egyptian blue pigment^14^ is just the result of a 0.90 eV shift induced by 
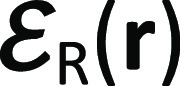
 on the highest d–d transition of the square-planar CuO_4_^6–^ chromophore in CaCuSi_4_O_10_.

An insight into the Δ gap between a_1g_(∼3*z*^2^ – *r*^2^) and b_1g_(∼*x*^2^ – *y*^2^) levels of tetragonal complexes thus requires taking into account both the intrinsic, Δ_int_, and extrinsic, Δ_ext_, contributions. For the sake of clarity, the values of both contributions derived for systems with tetragonal CuF_6_^4–^ units are displayed in Table 1. It is worth noting that the existence of an internal electric field allows one to understand why the hole in KAlCuF_6_^45^ and K_2_ZnF_4_:Cu^2+^^52^ is in the a_1g_(∼3*z*^2^ – *r*^2^) orbital in contrast to Cu^2+^ doped into the cubic perovskites KZnF_3_ or CsCdF_3_ where it resides in b_1g_(∼*x*^2^ – *y*^2^) due to a static Jahn–Teller effect.^30,32^ Indeed, for d^9^ ions under an initial octahedral symmetry, the existence of a static Jahn–Teller effect usually leads to elongated complexes^34,35^ with the exception of CaO:Ni^+^, a matter dealt with in refs (59) and (60). The extrinsic contribution arising from a tetragonal internal field in K_2_ZnF_4_:Cu^2+^ and Ba_2_ZnF_4_:Cu^2+^ also explains why |Δ| has been detected^46,52^ in the 0.6–1 eV region, whereas for KZnF_3_:Cu^2+^ it should be below 0.5 eV,^31,52^ a fact that concurs with the cubic symmetry of the host lattice. Bearing these facts in mind, the chemical bonding inside the complex essentially influences the intrinsic component, Δ_int_, of the total gap.

**Table 1:** Calculated Intrinsic, Δ_int_, and Extrinsic, Δ_ext_, Contributions to the a_1g_(∼3*z*^2^ – *r*^2^)–b_1g_(∼*x*^2^ – *y*^2^) Gap, Δ, for Systems Displaying Tetragonal CuF_6_^4–^ Units, with Cu^2+^–F^–^ Distances *R*_eq_ and *R*_ax_ (in Å Units)^a^

system	*R*_ax_	*R*_eq_	Δ_int_	Δ_ext_	Δ = Δ_int_ + Δ_ext_	|Δ(exp)|	refs
KAlCuF_6_	1.88	2.12	0.68	0.23	0.91	0.83	(42,44,45)
CuFAsF_6_	1.84	2.17	0.83	0.36	1.19		(44,45)
K_2_ZnF_4_:Cu^2+^	1.93	2.04	0.33	0.28	0.61	∼0.70	(24,46,52)
Ba_2_ZnF_4_:Cu^2+^	1.89	2.07	0.53	0.40	0.93	0.80	(24,47)
KZnF_3_:Cu^2+^	2.10	1.97	–0.36	∼0	–0.36	<0.5	(24,31,52)

a All energies are given in eV units. When *R*_ax_ < *R*_eq_, the ground state has a hole in a_1g_(∼3*z*^2^ – *r*^2^) and the extrinsic contribution tends to enhance the value of the intrinsic one. By contrast, for Cu^2+^ doped into the cubic perovskite KZnF_3_, displaying an elongated equilibrium geometry, the hole in the ground state lies in b_1g_(∼*x*^2^ – *y*^2^) and thus the sign of Δ_int_ is negative. The total calculated gap, Δ = Δ_int_ + Δ_ext_, is compared to available experimental data. The values of *R*_ax_ and *R*_eq_ metal–ligand distances are taken from experimental data for pure compounds and from calculations for systems where Cu^2+^ enters as an impurity.

**Figure 1:**
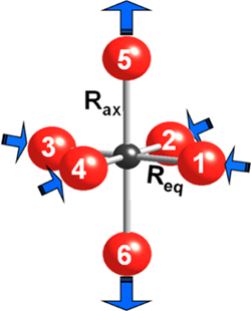
Description of a tetragonally elongated CuF_6_^4–^ complex.

Let us consider an isolated MX_6_ unit with a small tetragonal distortion depicted in Figure 1. This condition just implies that, if *R*_ax_ and *R*_eq_ are the two metal–ligand distances, with a mean value *R*_m_ = (*R*_ax_ + 2*R*_eq_)/3, it must be verified that
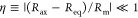
2where η reflects the tetragonal distortion from an octahedral MX_6_ complex with a metal–ligand distance equal to the mean value *R*_m_.

The condition given by eq 1 is well followed by all systems of Table 1 involving CuF_6_^4–^ units, where η < 0.16. As Δ_int_ should always be zero when *R*_ax_ = *R*_eq_; then it is function of *R*_ax_ – *R*_eq_ and can be written in a good first approximation as^45,49,52^
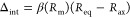
3where the β(*R*_m_) quantity only depends on the *R*_m_ value. Previous studies on systems^45,49,52^ with tetragonal CuF_6_^4–^ units lead to a value β ≅ 2.7 eV/Å when *R*_m_ ≅ 2.05 Å. It should be remarked that eq 3 is valid for systems displaying a static Jahn–Teller effect (like KZnF_3_:Cu^2+^) as well as for those whose ground state is determined by the internal electric field,^45^ such as it happens for CuFAsF_6_ or K_2_ZnF_4_:Cu^2+^. Therefore, the β quantity is *common* to all systems with tetragonal CuF_6_^4–^ units provided *R*_m_ ≅ 2.05 Å.

In the rough CF approach, where ligands are treated as point charges, the electrostatic potential due to ligands, *V*_M_, *around* the central cation (placed at **r** = **0**) involves two contributions^61^
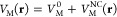
4Here, *V*_M_^0^ is independent of the electronic coordinate, **r**, but plays a key role for placing the energy of 3d levels of Cu^2+^ above that of 2p(F) ligand levels.^61^ Thus, within the CF framework, the non-constant contribution *V*_M_^NC^(**r**) is the only one responsible for the splitting Δ_int_ when *R*_eq_ ≠ *R*_ax_. Accordingly, the gap, Δ_int_(CF), for an isolated CuF_6_^4–^ unit, derived from the simple CF approach, is given by^17^
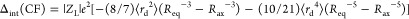
5where ⟨*r*_d_^2^⟩ = 1.044 a.u. and ⟨*r*_d_^4^⟩ = 2.674 a.u. correspond to *free* Cu^2+^ ions^62^ and *Z*_L_ is the ligand charge. Using these values, *R*_m_ = 2.05 Å, and even fully neglecting covalency, assuming *Z*_L_ = −1, we obtain from eqs 3 and 5 that β(CF) = 0.90 eV/Å, which is three times smaller than the value β ≅ 2.7 eV/Å corresponding to CuF_6_^4–^ at *R*_m_ = 2.05 Å. This comparison strongly suggests that the intrinsic contribution, Δ_int_, is greatly due to the covalent bonding inside the CuF_6_^4–^ unit. In the same way, the experimental 10*Dq* value of octahedral TM complexes is much higher than that calculated under the CF approach provided the right ⟨*r*_d_^4^⟩ quantity is employed.^54,61^

For understanding the role played by chemical bonding upon Δ_int_, it is useful to explore how the energy levels are modified as far as we increase the size of the basis set, following an approach first proposed by Löwdin.^63,64^ In the present case, let us start with a basis set which includes only the two purely d-wavefunctions of the central cation that are degenerate under an octahedral symmetry |*d*_a_⟩ = |3*z*^2^ – *r*^2^⟩ and |*d*_b_⟩ = |*x*^2^ – *y*^2^⟩. Although in this first step there is no chemical bonding in the two a_1g_(∼3*z*^2^ – *r*^2^) and b_1g_(∼*x*^2^ – *y*^2^) levels, their energy is significantly raised by the repulsive interaction of electrons with the negatively charged ligands involved in the *V*_M_^0^ term of eq 4. Also, in this step, the associated energies, *E*_a_ and *E*_b_, of a_1g_(∼3*z*^2^ – *r*^2^) and b_1g_(∼*x*^2^ – *y*^2^) levels can be written as
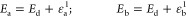
6where *E*_d_ corresponds to the octahedral situation (*R*_ax_ = *R*_eq_), while the corrections ε_a_^1^ and ε_b_^1^ are not strictly equal due to small CF effects under tetragonal symmetry. Indeed, in this first step Δ_int_ ≅ ε_b_^1^ – ε_a_^1^ whose expression is just given by eq 5.

In a second step, the ligand 2p and 2s wavefunctions are introduced in the basis set and then there is a change of both energy and shape of wavefunctions following the allowed 3d(TM)–2p(F) and 3d(TM)–2s(F) admixtures and the formation of antibonding orbitals. The linear combinations of 2p_σ_ and 2s wavefunctions involving axial and equatorial ligands and transforming like a_1g_ and b_1g_ are shown on Table 2. In the case of the a_1g_ irreducible representation, there are two contributions termed as χ_j_^eq^(*a*) and χ_j_^ax^(*a*) (j = pσ, s), which can be mixed with *d*_a_ = 3*z*^2^ – *r*^2^, while *d*_b_ = *x*^2^ – *y*^2^ can only be hybridized with the linear combinations χ_j_^eq^(b) (j = pσ, s) of equatorial ligands.

**Table 2:** Description of Antibonding Molecular Orbitals for Isolated Tetragonal CuF_6_^4–^ Units^a^

Cu^2+^	F ligands	χ_p_	χ_s_
3*z*^2^ – *r*^2^	equatorial	–1/2{p_σ_(1) + p_σ_(2) + p_σ_(3) + p_σ_(4)}	–1/2{s(1) + s(2) + s(3) + s(4)}
	axial	(1/√2){p_σ_(5) + p_σ_(6)}	(1/√2){s(5) + s(6)}
*x*^2^ – *y*^2^	equatorial	1/2{p_σ_(1) – p_σ_(2) + p_σ_(3) – p_σ_(4)}	1/2{s(1) – s(2) + s(3) – s(4)}

a χ_p_ and χ_s_ mean ligand wavefunctions hybridized with 3*z*^2^ – *r*^2^ and *x*^2^ – *y*^2^ orbitals of the central cation involving linear combinations of 2p(F) and 2s(F) orbitals, respectively. The positions of six ligand ions are shown in Figure 1.

Accordingly, in this second step, the normalized |a_1g_(∼3*z*^2^ – *r*^2^)⟩ and |b_1g_(∼*x*^2^ – *y*^2^)⟩ wavefunctions have the form
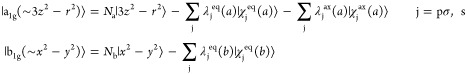
7

Although the λ_j_^eq^(*a*), λ_j_^ax^(*a*), and λ_j_^eq^(*b*) quantities are independent under *D*_4*h*_ symmetry (*R*_eq_ ≠ *R*_ax_), this is no longer true in the octahedral limit as they are related by the conditions
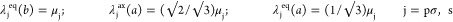
8

It is worth noting that although wavefunctions such as |*x*^2^ – *y*^2^⟩ and |χ_j_^eq^(*b*)⟩ (j = pσ, s) are not orthogonal, the associated overlap integrals *S*_pσ_ = ⟨*x*^2^ – *y*^2^|χ_pσ_^eq^(*b*)⟩ and *S*_s_ = ⟨*x*^2^ – *y*^2^|χ_s_^eq^(*b*)⟩ are both only of the order of 0.1 at equilibrium^65^ for MF_6_^4–^ complexes (M = Cu, Ni, Co, Fe). For this reason, the total electronic charges *q*_pσ_^eq^(*b*) and *q*_s_^eq^(*b*) transferred from the central cation to 2pσ and 2s orbitals of equatorial ligands in the antibonding |b_1g_(∼*x*^2^ – *y*^2^)⟩ orbital are reasonably given by
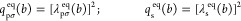
9

Similarly, the charges, *q*_j_^ax^(*a*) and *q*_j_^eq^(*a*) (j = pσ, s) transferred to axial and equatorial ligands in the antibonding |a_1g_(∼3*z*^2^ – *r*^2^)⟩ orbital can be approximated by
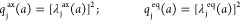
10

In the present step, the values of orbital energies and the 3d–2p and 3d–2s admixtures come from the solution of the secular equation
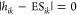
11

If we now work in second-order perturbations, the energy variations, ε_a_^2^ and ε_b_^2^, induced by chemical bonding upon the |a_1g_⟩ and |b_1g_⟩ orbitals can be approximated by
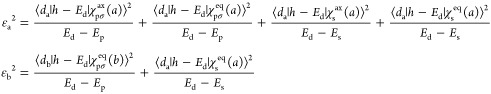
12Here, *E*_d_ – *E*_p_ and *E*_d_ – *E*_s_ stand for the separation between the 3d levels of the central cation and the 2p and 2s levels of ligands in the complex. From the present calculations for CuF_6_^4–^ at *R*_m_ = 2.05 Å, it is found that *E*_d_ – *E*_p_ ≅ 6 eV while *E*_d_ – *E*_s_ ≅ 26 eV.

Thus, if Δ_int_ is mainly governed by the different chemical bonding in |a_1g_⟩ and |b_1g_⟩ orbitals, then
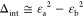
13

In the same vein, within the second-order perturbation approach, the covalency parameters λ_j_^eq^(*a*), λ_j_^ax^(*a*), and λ_j_^eq^(*b*) are given by
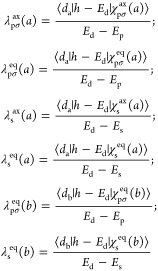
14

Thus, bearing eqs. 1, 7, 8, and 10–12 in mind, Δ_int_ can finally be related to the charges transferred to 2pσ and 2s ligand orbitals as follows
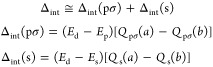
15where
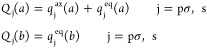
16

Therefore, according to eq 15, there are two contributions to the gap Δ_int_ reflecting the bonding with 2pσ and 2s ligand orbitals. That gap should be zero when the CuF_6_^4–^ unit is perfectly octahedral (*R*_eq_ = *R*_ax_) and in fact eq 15, in conjunction with eqs 8–10, leads to Δ_int_ = 0 in such a limiting case. Furthermore, eq 15 stresses that the gap, Δ_int_, is associated with variations of ligand charges on passing from an octahedral situation (η = 0) to a tetragonal one where *R*_eq_ ≠ *R*_ax_, a view consistent with the general Hohenberg–Kohn theorem.^66^ Indeed, the change of octahedral to tetragonal symmetry implies modifications of the electron–nuclei interactions (the so-called *external potential* in DFT^66^) and necessarily of the associated electronic density. This change in the electronic density is then reflected on variations of ligand charges.

As the present analysis is based on a second-order perturbation approach, its validity requires that in a level like |b_1g_(∼*x*^2^ – *y*^2^)⟩ the charges *q*_pσ_^eq^(*b*) and *q*_s_^eq^(*b*) transferred to ligands are clearly smaller that the unity. The condition *q*_pσ_^eq^(*b*) ≪ 1 is better accomplished for fluoride than chloride or bromide complexes due to the higher electronegativity of fluorine (3.9) when compared to that of Cl (3.0) or Br (2.8). By contrast, the condition *q*_s_^eq^(*b*) ≪ 1 is much better fulfilled for all kinds of complexes due to the deep character of 2s(F), 3s(Cl), or 4s(Br) levels of free atoms.^61^ For instance, the present calculations for CuF_6_^4–^ units, discussed in the next section, give *q*_pσ_^eq^(*b*) < 0.23, while a much lower value, *q*_s_^eq^(*b*) < 0.06, is obtained for the charge transferred to 2s orbitals. This relevant fact also stresses the perturbative character of the 3d–2s admixture.

The present approach focused on the Δ_int_ gap of tetragonal units has also been employed for understanding the intrinsic and dominant contribution to the 10*Dq* value of octahedral complexes and its dependence upon the metal–ligand distance.^67^ Interestingly, in the case of octahedral CrX_6_^3–^ complexes (X = F, Cl, Br, I), it has been found^67^ that the charge transferred to the valence ns level of ligands (*n* = 2, 3, 4, and 5 for F, Cl, Br, and I, respectively) in the antibonding e_g_(σ) orbital is always smaller than 0.1.

For the sake of clarity, when η ≠ 0 the Cu-wavefunction of the |a_1g_⟩ orbital is not a purely 3*z*^2^ – *r*^2^ orbital as it involves a small admixture (∼1%) of 4s(Cu). For elongated complexes, that admixture tends to enhance the electronic density of axial ligands, a matter discussed in ref (68). For obtaining such a 3d(Cu)–4s(Cu) hybridization in the present scheme, it is however necessary to go beyond the second-order approach.

## 4 Results and Discussion

### 4.1 Ligand Charges and Δ Gap for Isolated CuF_6_^4–^ Units: Influence of the Tetragonal Distortion

DFT calculations on the isolated CuF_6_^4–^ unit have been carried out using the transition state configuration a_1g_^1.5^b_1g_^1.5^, varying the equatorial and axial metal–ligand distances but maintaining the mean distance *R*_m_ = 2.05 Å. This allows one to calculate the Δ_int_ gap simply by means of the Janak theorem^66^ and to determine the charges transferred to 2pσ and 2s ligand orbitals for both a_1g_ and b_1g_ levels. Indeed, the use of the *average* a_1g_^1.5^b_1g_^1.5^ configuration allows one to establish a reasonable link with the analysis carried out in Section 3 based on orbitals associated with a given electronic configuration.

The main results are collected in Table 3. The calculated Δ_int_ values in Table 3 are consistent with the law embodied in eq 3 showing, in particular, that Δ_int_ just changes sign on passing from *R*_eq_ – *R*_ax_ = 0.15 Å to *R*_eq_ – *R*_ax_ = −0.15 Å. A value β = 2.8 eV/Å for *R*_m_ = 2.05 Å is derived from the present calculations.

**Table 3:** Charges Transferred to Ligands Calculated for an Isolated CuF_6_^4–^ Complex at Different Equatorial and Axial Metal–Ligand Distances, *R*_eq_ and *R*_ax_, But *Keeping* the Same Value of the Mean Distance *R*_m_ = (*R*_ax_ + 2*R*_eq_)/3 = 2.05 Å^a^

	*R*_eq_ = 2.05 Å, *R*_ax_ = 2.05 Å	*R*_eq_ = 2.00 Å, *R*_ax_ = 2.15 Å	*R*_eq_ = 1.95 Å, *R*_ax_ = 2.25 Å	*R*_eq_ = 2.10 Å, *R*_ax_ = 1.95 Å
*q*_pσ_^ax^(*a*)	0.148	0.148	0.147	0.142
*q*_pσ_^eq^(*a*)	0.074	0.068	0.062	0.082
*q*_pσ_^eq^(*b*)	0.222	0.225	0.227	0.219
*Q*_pσ_(*a*) – *Q*_pσ_(*b*)	0	–0.009	–0.018	0.005
Δ_int_(pσ) (eV)	0	–0.06	–0.12	0.03
q_s_^ax^(*a*)	0.023	0.026	0.028	0.025
q_s_^eq^(*a*)	0.010	0.002	0	0.016
q_s_^eq^(*b*)	0.033	0.041	0.051	0.025
*Q*_s_(*a*) – *Q*_s_(*b*)	0	–0.013	–0.033	0.016
Δ_int_(s) (eV)	0	–0.34	–0.86	0.42
Δ_int_ (eV)	0	–0.42	–0.84	0.42

a Results are reported for both a_1g_(∼3*z*^2^ – *r*^2^) and b_1g_(∼*x*^2^ – *y*^2^) orbitals. The contributions Δ_int_(pσ) and Δ_int_(s) to the energy gap Δ_int_, derived from the *Q*_pσ_(*a*) – *Q*_pσ_(*b*) and *Q*_s_(*a*) – Q_s_(*b*) quantities, are also shown. It can be noted that the value of Δ_int_(pσ) + Δ_int_(s) is close to the gap, Δ_int_, obtained in a DFT calculation for every value of *R*_eq_ and *R*_ax_.

As it is shown in Table 3, the charges transferred to 2pσ orbitals are, as expected, higher than those corresponding to 2s orbitals. However, when the tetragonality increases, the relative variation of q_pσ_^eq^(*b*) or q_pσ_^eq^(*a*) quantities is much smaller than that of q_s_^eq^(*b*) or q_s_^eq^(*a*) associated with 2s(F) orbitals. For instance, on passing from the octahedral situation (*R*_eq_ = *R*_ax_ = 2.05 Å) to *R*_eq_ = 1.95 Å and *R*_ax_ = 2.25 Å, q_s_^eq^(*b*) increases by 55% while q_pσ_^eq^(*b*) changes only by 2% and thus it remains nearly constant.

The quantities q_s_^eq^(*b*) and q_pσ_^eq^(*b*) are deeply related to the isotropic (*A*_s_) and anisotropic (*A*_p_) superhyperfine constants, respectively, for *elongated* CuF_6_^4–^ units formed in Cu^2+^-doped fluoroperovskites as a result of a static Jahn–Teller effect.^30,32^ Low-temperature electron paramagnetic resonance data indicate that whereas for CsCdF_3_:Cu^2+^*A*_s_ = 160 (5) MHz,^32^ it clearly increases up to *A*_s_ = 183 (5) MHz^30^ for KZnF_3_:Cu^2+^. By contrast, the measured values *A*_p_ = 76 (5) MHz for CsCdF_3_:Cu^2+^ and *A*_p_ = 68 (5) MHz for KZnF_3_:Cu^2+^ are coincident within experimental uncertainties. This fact is consistent with results for elongated NiF_6_^5–^ species in different fluoroperovskites^72,73^ involving the 3d^9^ ion Ni^+^, where *A*_s_ and *q*_s_^eq^(*b*) are highly sensitive to the actual value of *R*_eq_ but not *A*_p_ or *q*_pσ_^eq^(*b*). Indeed, whereas *A*_p_ changes only by 3% along the series of fluoroperovskites, the variation of *A*_s_ is 1 order of magnitude higher (30%).

In the same vein as for octahedral NiF_6_^4–^, MnF_6_^4–^, or FeF_6_^3–^ units in cubic fluoroperovskites,^54,74−79^ both *A*_s_ and *q*_s_ quantities, corresponding to the e_g_(σ) orbital, are strongly dependent upon the metal–ligand distance, while *A*_p_ is much less sensitive.

These facts already suggest that, according to eq 15, the gap Δ_int_ is mainly due to the Δ_int_(s) contribution reflecting changes in the 3d–2s admixture when the tetragonality increases. This idea is certainly reinforced looking at results of present calculations embodied in Table 3. Indeed, such results prove that the obtained Δ_int_(s) contribution essentially accounts for the calculated gap Δ_int_ at different values of *R*_eq_ and *R*_ax_ distances. For instance, for *R*_eq_ = 1.95 Å and *R*_ax_ = 2.25 Å, the results of Table 3 give Δ_int_(pσ) = −0.10 eV and Δ_int_(s) = −0.86 eV. Therefore, comparing these values with the figure Δ_int_ = −0.84 eV derived from DFT calculations, we can conclude that such a gap is greatly the result of variations of the 3d–2s admixture with the tetragonality. Although this conclusion may be surprising, we can note that, from results of Table 3 for *R*_eq_ = 1.95 Å and *R*_ax_ = 2.25 Å, it is verified that
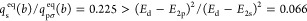
17just implying that
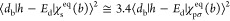
18

Thus, the coupling of the 2s-ligand wavefunction, χ_s_^eq^(*b*), with |*d*_b_⟩ = |*x*^2^ – *y*^2^⟩ is a little stronger than that for the 2p-wavefunction, χ_p_^eq^(*b*). This conclusion is qualitatively consistent with the Wolfsberg–Helmholz guess^80^ used before the arrival of ab initio calculations.

Bearing eq 3 in mind, we have also explored the dependence of Δ_int_ and the β quantity upon the average value of the metal–ligand distance, *R*_m_. Varying *R*_m_ in the range 1.95–2.05 Å we have found that Δ_int_ and β are sensitive to the value of *R*_m_ according to the law
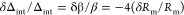
19

We have verified that the increase of Δ_int_ and β when *R*_m_ decreases is also followed by an increase of the *Q*_s_(*a*) – *Q*_s_(*b*) quantity while the contribution of *Q*_pσ_(*a*) – *Q*_pσ_(*b*) is again much less sensitive to the change of *R*_m_. This situation is thus akin to that described in Table 3.

### 4.2 Variation of 10*Dq* with the Metal–Ligand Distance for Octahedral Complexes

Bearing the present results and those previously obtained^67^ on *O*_*h*_ complexes in mind, we want now to explain quantitatively the origin of the dependence of 10*Dq* on the metal–ligand distance, *R*.

Experimental values for a variety of octahedral complexes^54,61,67,79^ lead to an *R* dependence of the intrinsic contribution to 10*Dq*, (10*Dq*)_int_, given by^54^
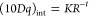
20where the exponent *t* usually lies in the 4–6 range and thus it is close to the value *t* = 5 provided by CF theory.^17^

According to previous results, (10*Dq*)_int_ can reasonably be approximated by^67^
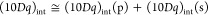
21where the ratio α = (10*Dq*)_int_(s)/(10*Dq*)_int_ has been found to be around 0.65^67^ for the series of CrX_6_^3–^ units (X = F, Cl, Br, I). Similarly to results of Section 4.1, the changes of (10*Dq*)_int_ due to *R* variations are essentially driven by the (10*Dq*)_int_(s) contribution, reflecting the dependence of the *q*_s_ charge of 2s, 3s, 4s, or 5s ligand orbitals on the metal–ligand distance.

Thus, writing
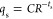
22and considering small *R* variations (δ*R* ≪ *R*), the following quantitative relation among *t*, *t*_s_, and α comes out
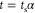
23

Values of the exponent *t*_s_ in the 6.5–8.5 range have been derived for doped cubic fluorides^54,67,76,79^ and are responsible for the high sensitivity of the isotropic superhyperfine constant, *A*_s_, to *R* variations well observed experimentally.^54,74−79^

For Mn^2+^-doped cubic fluoroperovskites, a value *t*_s_ = 8 has been obtained,^76^ while from the parallel study of optical spectra,^81^*t* = 4.7 is found. These values are thus consistent with eq 23 and α ≈ 0.6.

Despite this fact and the early work by Sugano and Shulman,^16,17^ proving that (10*Dq*)_int_ essentially reflects the different covalent bonding in e_g_(σ) and t_2g_(π) levels, experimental values of the exponent t close to 5 are still taken as a support to the validity of CF theory.^40^

The sensitivity of 10*Dq* to *R* variations has a useful application for changing the shape of the fluorescence band in fluorides doped with Cr^3+^. Indeed, while the emission spectrum at ambient pressure of both Cr^3+^-doped KZnF_3_ and K_2_NaGaF_6_ lattices is a broad band arising from the ^4^T_2_ excited state, a sharp ruby-like spectrum coming from a ^2^E first excited state is detected for pressures smaller than 15 GPa.^82,83^

### 4.3 Key Role of Deep 2s(F) Orbitals in Chemical Bonding and the 2p(F)–2s(F) Gap: Microscopic Origin

**Figure 2:**
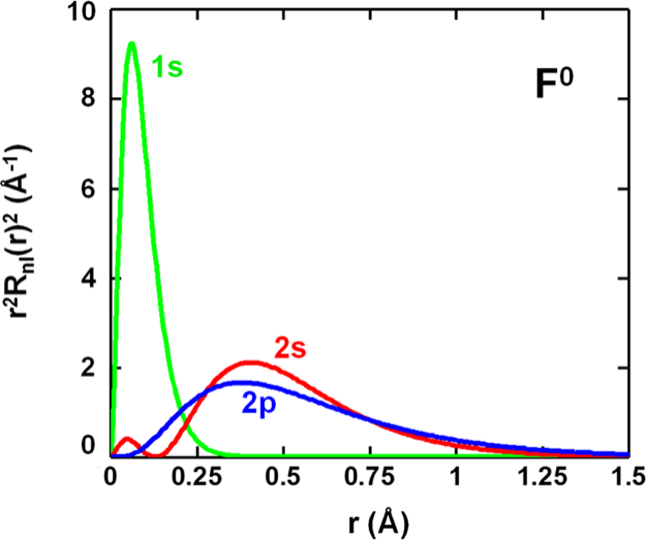
Radial probability densities *P*_j_(*r*) = *r*^2^*R*_j_^2^(*r*) (j = 1s, 2s, 2p) corresponding to free fluorine atom calculated by means of the atomic wavefunctions of Bunge et al.^29^

The big separation, ε(2p) – ε(2s) ≅ 23 eV, between 2p and 2s levels of both free fluorine atom and the negative F^–^ ion,^27−29^ cannot be ascribed to a different extent of such orbitals. If we denote by *R*_2p_(*r*) and *R*_2s_(*r*) the radial functions of 2p and 2s orbitals and by *R*_1s_(*r*) that of the inner 1s orbital, in Figure 2 are depicted the radial probability densities *P*_j_(*r*) = *r*^2^*R*_j_^2^(*r*) (j = 1s, 2s, 2p) corresponding to free fluorine atom. While the maximum of *P*_1s_(*r*) is reached for *r*_1s_ = 0.06 Å, those for 2p and 2s orbitals both appear at higher distances but are very close. Indeed, as shown in Figure 2, the maximum of *P*_2p_(*r*) is at *r*_2p_ = 0.36 Å and that of *P*_2s_(*r*) at *r*_2s_ = 0.40 Å. It should be noted however that, when *r* > 0.75 Å, *P*_2p_(*r*) is always a bit higher than *P*_2s_(*r*). This situation is consistent with the calculated overlap integrals *S*_pσ_ and *S*_s_ for a series of octahedral MF_6_ complexes^65^ (M = Ni^2+^, Co^2+^, Mn^2+^, Fe^3+^, Cr^3+^, Mn^4+^). Indeed, at equilibrium distances, *S*_pσ_ and *S*_s_ are both around 0.1 although *S*_pσ_ is a little higher than *S*_s_. Nevertheless, due to the longer tail of the 2p orbital when compared to the 2s wavefunction (Figure 2), the dependence of *S*_s_ upon the metal–ligand distance is stronger^73^ than that of *S*_pσ_.

The behavior of radial 2p and 2s wavefunctions depicted in Figure 2 is also consistent with the fact that the matrix elements ⟨*d*_b_|*h* – *E*_d_|χ_pσ_^eq^⟩ and ⟨*d*_b_|*h* – *E*_d_|χ_s_^eq^⟩ involved in eq 18 are comparable. Indeed, they just reflect that both functions look rather similar when *r* > 0.5 Å.

Thus, if the extent of 2p and 2s orbitals is comparable, it is now necessary to understand why ε(2p) – ε(2s) ≅ 23 eV for fluorine, while that gap is strictly equal to zero for the hydrogen atom and hydrogenic ions such as He^+^ or Li^2+^.

It should first be stressed that the degeneracy between 2p and 2s orbitals in hydrogen is far from being accidental. In fact, it is the result of an invariant quantity, which appears however only when the potential energy, *U*(*r*), seen by the electron is strictly Coulombian^84^ and thus has the form
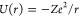
26at every distance *r* from the nucleus.

When this condition is fulfilled, in addition to the angular momentum, **L**, the so-called Runge–Lenz operator, **A**, also commutes with the Hamiltonian of the problem.^84^ The expression of that operator is given by
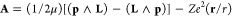
27where μ means the reduced mass of the hydrogen atom. The **A** operator connects the radial *R*_2s_(**r**) and *R*_2p_(**r**) wavefunctions and thus implies that the corresponding levels are to be degenerate. This operator was used by Pauli^85^ for solving the energy spectrum of the hydrogen atom in the framework of the matrix quantum mechanics by Heisenberg and Born.

The Runge–Lenz vector also plays a relevant role in studying the motion of planets around the sun. Its invariance implies that the position of the perihelion remains constant in time.^86^

In an atom different from H or ions such as He^+^ or Li^2+^, the self-consistent potential felt by a valence electron is not described by eq 26 in the whole range of distances to the nucleus as the net charge, *Ze*, seen by the electron depends on the *r* value. Therefore, for the fluorine atom, when *r* ≪ *r*_1s_, *Z* ≅ 9, whereas when *r*_1s_ < *r* < *r*_2s_, *Z* would be around 7 due to the screening by two inner electrons.

Bearing these facts in mind, the origin of the big separation between 2p and 2s levels in fluorine atom stems from the different behavior of the wavefunctions in the internal *r* < 0.1 Å region. As shown in Figure 2, *P*_2s_(*r*) has a small maximum at *r*_M_ = 0.04 Å with *P*_2s_(*r*_M_) = 0.4 Å^–1^. By contrast, *P*_2p_(*r*) is essentially zero in the 0 < *r* < 0.1 Å region as a result of the *l*(*l* + 1)/*r*^2^ term in the radial equation making that *R*_2p_(0) = 0 but *R*_2s_(0) ≠ 0. Thus, the 2s charge *P*_2s_(*r*_M_)Δ*r* = 0.02*e* for Δ*r* = 0.05 Å implies an energy gain for the 2s orbital with respect to the 2p one in that internal region, which can be estimated to be ∼20 eV using the virial theorem and *Z* = 7. It is worth noting that, if the 2p–2s separation in F mainly arises from the distinct behavior of both wavefunctions in the internal region, it is also consistent with a ε(2p) – ε(2s) value for F^–^ that is only 5% higher than for the fluorine atom.^27,61^ In the same vein, the value of ε(*n*p) – ε(*n*s) for Cl^–^ (*n* = 3) and Br^–^ (*n* = 4) ions is only 2% higher than that for the corresponding free atom.^27,61^

These considerations thus account for the big ε(2p) – ε(2s) value for fluorine and explain the fact that *q*_s_^eq^(*b*) ≪ *q*_pσ_^eq^(*b*). Moreover, due to the similar extent of the radial 2p and 2s wavefunctions when *r* > 0.5 Å, we can understand that the bonding with deeper 2s(F) orbitals is not negligible.

Nevertheless, it is surprising that the value of the Δ_int_ gap essentially arises from the 3d–2s admixture rather than from the 3d–2p one despite *q*_s_^eq^(*b*) ≪ *q*_pσ_^eq^(*b*). However, from eq 15 and the results embodied in Table 3, this surprising conclusion is fully consistent with the near independence of *q*_pσ_^eq^(*b*) charges on the *R*_ax_ – *R*_eq_ value describing the tetragonal distortion. By contrast, *q*_s_^eq^(*b*) increases by 55% on passing from *R*_eq_ = *R*_ax_ = 2.05 Å to the *D*_4*h*_ geometry corresponding to *R*_eq_ = 1.95 Å and *R*_ax_ = 2.25 Å (Table 3).

This situation is thus akin to that encountered for the antibonding e_g_ orbitals of octahedral complexes.^54,79^ Therefore, for the e_g_(*x*^2^ – *y*^2^) orbital, *q*_pσ_^eq^ is again found to be higher than *q*_s_^eq^ but the dependence of *q*_s_^eq^ on the metal ligand distance, *R*, is much stronger than that of *q*_pσ_^eq^.

This important result has been explained^54,73,79^ considering that λ_pσ_^eq^(e_g_) depends on the ratio ⟨*d*(*x*^2^ – *y*^2^)|*h* – *E*_d_|χ_pσ_^eq^⟩/(*E*_d_ – *E*_p_). Accordingly, when *R* is reduced, the quantity |⟨*d*(*x*^2^ – *y*^2^)|*h* – *E*_d_|χ_pσ_^eq^⟩| increases roughly following the corresponding overlap integral *S*_pσ_. However, this increase is compensated by the additional rise of the charge-transfer excitation *E*_d_ – *E*_p_ due to the lessening of the metal–ligand distance on an isolated 3d complex.^54,73,87^ By contrast, in the case of the admixture with the deeper 2s(F) orbital, the variation of *q*_s_^eq^ with the distance essentially reflects that of [*S*_s_(*R*)]^2^. Examples of this behavior are shown in refs.^73,76,79^

## 5 Final Remarks

The present work highlights that the relation between spectroscopic data of TM compounds with the chemical bonding can be very subtle.

When in an isolated CuF_6_^4–^ complex, we move from an initial octahedral situation (η = 0) to a tetragonal one with η ≠ 0 the energy of eigenstates and thus Δ_int_ are modified. There are two sources of that change: (a) the dependence on η of the Hamiltonian and (b) the additional dependence on the distortion of the associated wavefunctions. The present analysis supports that the main contribution to Δ_int_ arises from the variations undergone by b_1g_(∼*x*^2^ – *y*^2^) and a_1g_(∼3*z*^2^ – *r*^2^) wavefunctions when η is modified and thus the center of the gravity theorem^88^ cannot be applied. Furthermore, Δ_int_ is essentially associated with the variations experienced by the 2s(F) charge with η because the 2pσ(F) charge is nearly independent of the tetragonal distortion.

The present ideas can also be useful for understanding 3d complexes where fluorine is replaced by other halides or oxygen as ligand. Indeed, for these ligands, the ε(*n*p) – ε(*n*s) gap is also significant and lies in the 14–18 eV range.^61^ Taking as a guide the case of CdCl_2_:Cu^2+^, the tetragonal splitting, Δ_int_, has been measured^89^ to be equal to −0.79 eV as a result of a static Jahn–Teller effect, leading to an elongated octahedral geometry. As there are no available data on the equilibrium geometry of the CuCl_6_^4–^ unit in CdCl_2_, we have derived it through first-principles calculations giving *R*_eq_ = 2.33 Å and *R*_ax_ = 2.63 Å. On this basis, we obtain for CuCl_6_^4–^ in CdCl_2_ a value β = 2.6 eV/Å that is comparable to that reported in Section 4.1 for the isolated CuF_6_^4–^ unit. Furthermore, using these calculated *R*_eq_ and *R*_ax_ values in the CF expression for Δ_int_ given in eq 5, we obtain for CdCl_2_:Cu^2+^ a value Δ_int_(CF) = −0.12 eV, thus stressing the inadequacy of the CF approach.

From results of Section 4.1 the gap, Δ_int_, increases significantly upon applied pressures. This fact can be of interest in the realm of superconductor oxocuprates where the transition temperature, *T*_c_, is related^53^ to the value of such a gap.

Although the ground state of MnF_6_^4–^ and CrF_6_^3–^ units in cubic lattices is orbitally singlet, this is no longer true for T_1g_ and T_2g_ excited states^90^ where there is a coupling with the Jahn–Teller mode, e_g_, well seen through the progressions in luminescence spectra.^91^ From calculations carried out on MnF_6_^4–^, Δ_int_ = 0.147 eV for *R*_ax_ – *R*_eq_ = −0.06 Å was obtained,^90^ thus implying β = 2.46 eV/Å. This figure is thus similar to that derived for the ground state of tetragonal CuF_6_^4–^ units that also involves a divalent cation.

Electronic levels lying far from the HOMO play also an important role in the realm of structural instabilities.^92^ Therefore, due to the admixture of the ground with excited states via the electron-vibration coupling, the NH_3_ molecule is non-planar^93−95^ and an orthorhombic distortion appears in A_2_CuF_4_ (A = K, Na)^4,9,49^ and NH_4_Cl:CuCl_4_(H_2_O)_2_^2–^^51^ but not in NH_4_Cl:CuCl_4_(NH_3_)_2_^2–^.^96^ In the same vein, isolated Mn^+^ ions in KCl:Mn^+^^97^ or Cu^2+^ in SrCl_2_:Cu^2+^^54^ move spontaneously from the cubic site to an off-center position, a situation not found in BaTiO_3_ for an *isolated* Ti^4+^ ion, stressing that ferroelectricity involves a *cooperative* distortion of all Ti^4+^ ions.^97^ Interestingly, the value of the excitation involved in the instability of the ammonia molecule goes up to 12 eV^94,95^ although it is usually smaller for other systems^51^ such as NH_4_Cl:CuCl_4_(H_2_O)_2_^2–^.
